# Effects of Excluding Those Who Report Having “Syndomitis” or “Chekalism” on Data Quality: Longitudinal Health Survey of a Sample From Amazon’s Mechanical Turk

**DOI:** 10.2196/46421

**Published:** 2023-08-04

**Authors:** Ron D Hays, Nabeel Qureshi, Patricia M Herman, Anthony Rodriguez, Arie Kapteyn, Maria Orlando Edelen

**Affiliations:** 1 Division of General Internal Medicine and Health Services Research Department of Medicine University of California Los Angeles, CA United States; 2 Behavioral and Policy Sciences RAND Corporation Santa Monica, CA United States; 3 Behavioral and Policy Sciences RAND Corporation Boston, MA United States; 4 Center for Economic and Social Research University of Southern California Los Angeles, CA United States; 5 Patient Reported Outcomes, Value and Experience (PROVE) Center Department of Surgery Brigham and Women’s Hospital Boston, MA United States

**Keywords:** misrepresentation, survey, data quality, MTurk, Amazon Mechanical Turk

## Abstract

**Background:**

Researchers have implemented multiple approaches to increase data quality from existing web-based panels such as Amazon’s Mechanical Turk (MTurk).

**Objective:**

This study extends prior work by examining improvements in data quality and effects on mean estimates of health status by excluding respondents who endorse 1 or both of 2 fake health conditions (“Syndomitis” and “Chekalism”).

**Methods:**

Survey data were collected in 2021 at baseline and 3 months later from MTurk study participants, aged 18 years or older, with an internet protocol address in the United States, and who had completed a minimum of 500 previous MTurk “human intelligence tasks.” We included questions about demographic characteristics, health conditions (including the 2 fake conditions), and the Patient Reported Outcomes Measurement Information System (PROMIS)-29+2 (version 2.1) preference–based score survey. The 3-month follow-up survey was only administered to those who reported having back pain and did not endorse a fake condition at baseline.

**Results:**

In total, 15% (996/6832) of the sample endorsed at least 1 of the 2 fake conditions at baseline. Those who endorsed a fake condition at baseline were more likely to identify as male, non-White, younger, report more health conditions, and take longer to complete the survey than those who did not endorse a fake condition. They also had substantially lower internal consistency reliability on the PROMIS-29+2 scales than those who did not endorse a fake condition: physical function (0.69 vs 0.89), pain interference (0.80 vs 0.94), fatigue (0.80 vs 0.92), depression (0.78 vs 0.92), anxiety (0.78 vs 0.90), sleep disturbance (−0.27 vs 0.84), ability to participate in social roles and activities (0.77 vs 0.92), and cognitive function (0.65 vs 0.77). The lack of reliability of the sleep disturbance scale for those endorsing a fake condition was because it includes both positively and negatively worded items. Those who reported a fake condition reported significantly worse self-reported health scores (except for sleep disturbance) than those who did not endorse a fake condition. Excluding those who endorsed a fake condition improved the overall mean PROMIS-29+2 (version 2.1) T-scores by 1-2 points and the PROMIS preference–based score by 0.04. Although they did not endorse a fake condition at baseline, 6% (n=59) of them endorsed at least 1 of them on the 3-month survey and they had lower PROMIS-29+2 score internal consistency reliability and worse mean scores on the 3-month survey than those who did not report having a fake condition. Based on these results, we estimate that 25% (1708/6832) of the MTurk respondents provided careless or dishonest responses.

**Conclusions:**

This study provides evidence that asking about fake health conditions can help to screen out respondents who may be dishonest or careless. We recommend this approach be used routinely in samples of members of MTurk.

## Introduction

The use of innovative methods to reach potential survey respondents (eg, internet panels, Facebook, and Pollfish) has increased because they are cost-effective, provide access to large and diverse samples quickly, and take less time than traditional mail and phone modes of data collection. Amazon’s Mechanical Turk (MTurk) is a crowdsourcing platform that includes a pool of “workers” willing to complete tasks for low levels of compensation [1]. The extent to which MTurk and other convenience-based samples are representative of the general population [2] or subgroups of the population [3] is a concern in many studies. Most MTurk participants are young, White, male, and highly educated, but report relatively poor mental health [4,5]. In addition to questions about representativeness, problems with data integrity among MTurk respondents have been identified [6]. Chandler et al [7] found relatively low reliability of data provided by MTurk respondents who scored poorly on a test of comprehension and ability to respond to questions. Ophir et al [8] reported that the estimated prevalence of depression was about 50% higher when inattentive responders were included.

Researchers have implemented a variety of approaches to increase data quality from existing web-based panels such as removing those who have an average item response of 1 second or less, adding screener questions before the main survey, doing IP address verification, and conducting test-retest comparisons on demographic variables [9,10]. This study extends the work of Qureshi et al [5], by examining improvements in data quality and effects on mean estimates of health status by excluding respondents who endorse either or both of 2 fake health conditions (“Syndomitis” and “Chekalism”).

## Methods

### Study Design

We developed web-based surveys and used the web-based platform CloudResearch (formerly TurkPrime) to field the survey in 2021 with MTurk participants [11]. Eligible study participants were 18 years or older with an IP address in the United States and had to have completed a minimum of 500 previous MTurk “human intelligence tasks” (surveys, writing product descriptions, coding, or identifying content in images or videos) with a successful completion rate of at least 95%. The 95% threshold was selected because it is associated with better response quality [12]. Additional quality control measures included not telling participants that the study was targeting individuals with back pain and deploying small batches of surveys hourly over several weeks to reduce selection bias. We also screened for excessive speediness in completing the survey (<1 second per item) but no one responded that quickly.

All participants provided electronic consent at the start of the survey. Those who completed a general health survey and reported currently having back pain were asked to complete a back pain survey. Those who completed the general health and back pain survey were paid US $3.50 for participation. Payments were determined by approximating the amount of time needed to complete the survey and offering the equivalent of the US federal minimum wage for completion of the general health survey and a slight bonus for completing the subsequent back pain survey. Individuals who reported having back pain and did not endorse a fake condition at baseline were provided the opportunity to complete a follow-up survey 3 months later.

### Ethics Approval

All procedures were reviewed and approved by the research team’s institutional review board (RAND Human Subjects Research Committee FWA00003425; IRB00000051) and conform to the principles in the Declaration of Helsinki.

### Survey

The survey included questions about demographic characteristics and health conditions. Thirteen bona fide health conditions were assessed: whether the participants have ever been told by a doctor or other health professional that they had (1) hypertension, (2) high cholesterol, (3) heart disease, (4) angina, (5) heart attack, (6) stroke, (7) asthma, (8) cancer, (9) diabetes, (10) chronic obstructive pulmonary disease, (11) arthritis, (12) anxiety disorder, and (13) depression. In addition, the survey asked respondents if they were ever told they had “Syndomitis” (a fake condition). Further, participants were asked if they currently have nine other bona fide conditions: (1) allergies or sinus trouble, (2) back pain, (3) sciatica, (4) neck pain, (5) trouble seeing, (6) dermatitis, (7) stomach trouble, (8) trouble hearing, and (9) trouble sleeping. They were also asked if they have “Chekalism” (a fake condition).

The Patient-Reported Outcomes Measurement Information System (PROMIS)-29+2 (version 2.1) preference-based score (PROPr) was also administered [13]. The PROMIS-29+2 (version 2.1) includes 7 multi-item scales with 4 items each (physical function, pain interference, fatigue, sleep disturbance, depression, anxiety, ability to participate in social roles and activities), a 2-item cognitive function scale, and a single 0-10 pain intensity item. All items within 7 of the multi-item scales are worded in the same direction (eg, represent better health) but 2 of the items in the sleep disturbance scale were worded in the direction of less disturbance and the other 2 items were worded to indicate more disturbance. In addition to scores for the 8 scales and the single pain intensity item, the PROMIS-29+2 (version 2.1) yields physical health and mental health summary scores and a PROPr [14,15].

### Analysis Plan

The response rate to the survey was calculated as the number of respondents who completed at least half the items divided by the number of individuals invited to participate in the survey. We compute at baseline and the 3-month follow-up, estimates of internal consistency reliability [16] product-moment correlations among scales, and mean scores for the PROMIS-29+2 (version 2.1) separately for those who did not versus did endorse a fake health condition. We hypothesized that those who endorse a fake condition provide less reliable information, have smaller correlations among scales, and mean scores reflect worse health than those who do not endorse a fake condition.

## Results

### Overview

The survey response rate was 50% (6832/13,608). In total, 15% (996/6832) of the sample endorsed 1 or both of the 2 fake conditions at baseline. Characteristics of those who did versus did not endorse a fake condition are shown in Table 1.

**Table 1:** Characteristics of Amazon Mechanical Turk respondents (N=6832) endorsing and those not endorsing a fake health condition on the baseline health survey.

Variable			Did not endorse fake health conditions (n=5836)		Endorsed fake health condition (n=996)
**Gender, n (%)**					
	Female	2685 (46)		329 (33)	
	Male	3093 (53)		667 (67)	
	Transgender or do not identify as female, male, or transgender	58 (1)		0 (0)	
Non-White, n (%)			1050 (18)		279 (28)
Age (years), mean (SD)			40 (12)		38 (10)
Number of health conditions, mean (SD)			4 (3)		15 (5)
Time to complete (minutes), mean (SD)			19 (12)		27 (13)

Those who endorsed a fake condition at baseline were more likely to identify as male, non-White, younger, report more health conditions, and take longer to complete the survey than those who did not endorse a fake condition. As noted above, those who endorsed a fake condition at baseline were not asked to complete a 3-month survey. Even though they did not endorse a fake condition at baseline, 6% (n=59) endorsed at least 1 of the fake conditions on the 3-month survey (n=972, 94% did not endorse a fake condition). Therefore, the estimated proportion of fakers in the sample is 25% (1708/6832; Multimedia Appendix 1), within the range of 20%-30% of detected fraud reported in other web-based studies [17].

### Baseline Survey PROMIS-29+2 (Version 2.1) Data Quality and Mean Scores

Internal consistency reliabilities for the PROMIS-29+2 (version 2.1) scales were uniformly larger at baseline for those who did not endorse a fake condition than for those who did (Table 2). The α coefficient for sleep disturbance (the scale with 2 items worded in the direction of less sleep disturbance and the other 2 worded in the opposite direction) was negative.

Consistent with the difference in reliability estimates, most of the product-moment correlations among the PROMIS-29 (version 2.1) scales were lower for those who endorsed a fake condition than for those who did not (Table 3).

Those who endorsed a fake condition had worse self-reported health scores for all scales except for the sleep disturbance scale. Excluding those who endorsed a fake condition changed the mean PROMIS-29+2 (version 2.1) T-scores (except sleep disturbance) by 1-2 points and the PROPr by 0.04 toward better self-reported health. The sleep disturbance scale means did not differ between those who endorsed versus did not endorse a fake condition because the former provided inconsistent answers to the positively and negatively worded items (Table 4). Because those who endorsed a fake health condition tended to report worse health, the standard deviations of the PROMIS-29+2 (version 2.1) scales tended to be smaller than those seen among those who did not endorse a fake health condition.

**Table 2:** Internal consistency reliability of Patient-Reported Outcomes Measurement Information System 29+2 (version 2.1) preference–based score scales on the baseline health survey.

Scale	Did not endorse fake health condition (n=5836)	Endorsed fake health condition (n=996)
Physical function	0.89	0.69
Pain interference	0.94	0.80
Fatigue	0.92	0.80
Depression	0.92	0.78
Anxiety	0.90	0.78
Sleep disturbance	0.84	−0.27
Ability to participate in social roles or activities	0.92	0.77
Cognitive function	0.77	0.65

**Table 3:** Correlations among Patient-Reported Outcomes Measurement Information System-29+2 (version 2.1) preference–based score scales on the baseline survey (did not endorse is below and endorsed fake health condition is above the diagonal).

	PF^a^	PIter^b^	PIten^c^	FAT^d^	DEP^e^	ANX^f^	SLPD^g^	SOC^h^	CF^i^
PF		−0.12	−0.14	−0.20	−0.21	−0.26	−0.11	0.15	−0.01
PIter	−0.72		0.26	0.73	0.60	0.56	0.01	−0.78	0.64^j^
PIten	−0.59	0.72		0.32	0.29	0.29	0.01	−0.27	0.22^j^
FAT	−0.47	0.54	0.48		0.72	0.66	0.06	−0.70	0.50^j^
DEP	−0.43	0.50	0.45	0.71		0.77	0.09	−0.68	0.40^j^
ANX	−0.43	0.51	0.46	0.70	0.82		0.05	−0.64	0.39^j^
SLPD	−0.30	0.37	0.37	0.61	0.53	0.52		−0.03	−0.17
SOC	0.64	−0.72	−0.56	−0.68	−0.66	−0.66	−0.49		−0.54^j^
CF	0.33	−0.31	−0.29	−0.30	−0.37	−0.37	−0.31	0.39	

^a^PF: physical function.

^b^PIter: pain interference.

^c^PIten: pain intensity.

^d^FAT: fatigue.

^e^DEP: depression.

^f^ANX: anxiety.

^g^SLPD: sleep disturbance.

^h^SOC: ability to participate in social roles and activities.

^i^CF: cognitive function.

^j^Correlation is in the “wrong” direction.

**Table 4:** PROMIS-29+2^a^ (version 2.1; PROPr^b^) scale means (SDs) on the baseline survey.

Scale	Did not endorse fake health conditions (n=5836), mean (SD)	Endorsed fake health condition (n=996), mean (SD)	Overall sample (N=6832), mean (SD)
Physical function	49 (8)	41 (5)	48 (8)
Pain interference	51 (9)	63 (5)	53 (10)
Pain intensity	52 (10)	64 (9)	54 (11)
Fatigue	50 (10)	58 (8)	51 (10)
Depression	53 (10)	63 (7)	54 (10)
Anxiety	54 (10)	63 (7)	56 (10)
Sleep disturbance	50 (9)	51 (4)	50 (9)
Ability social roles or activities	53 (9)	43 (7)	52 (10)
Cognitive function	50 (9)	47 (7)	49 (9)
PROMIS-29 physical health summary	49 (9)	40 (3)	48 (9)
PROMIS-29 mental health summary	50 (9)	39 (6)	48 (9)
PROPr	0.45 (0.25)	0.20 (0.10)	0.41 (0.25)

^a^PROMIS: Patient-Reported Outcomes Measurement Information System.

^b^PROPr: PROMIS preference-based score.

### Three-Month Survey PROMIS-29+2 (Version 2.1) Data Quality and Mean Scores

Differences between those who reported on the 3-month survey that they had versus did not have a fake condition were similar to what was observed on the baseline survey. Internal consistency reliabilities for the PROMIS-29+2 (version 2.1) scales were uniformly larger for those who did not endorse a fake condition than for those who did (Table 5).

As in the baseline survey, the α coefficient for sleep disturbance was negative among those who endorsed a fake condition because this subgroup answered all 4 questions similarly despite the wording of 2 items indicating less sleep disturbance and 2 items indicating more sleep disturbance. Most of the product-moment correlations among the PROMIS-29+2 (version 2.1) scales were smaller for those who endorsed a fake condition than for those who did not (Table 6).

As was the case at baseline, those who endorsed a fake condition at 3 months had significantly worse health scores for all scales except for the sleep disturbance scale where they provided inconsistent answers to the positively and negatively worded items (Table 7). Further, the PROMIS-29+2 (version 2.1) scale score standard deviations tended to be lower for those who endorsed a fake health condition.

**Table 5:** Internal consistency reliability of the Patient-Reported Outcomes Measurement Information System-29+2 (version 2.1) preference–based score scales on the 3-month survey.

Scale	Did not endorse fake health conditions (n=972)	Endorsed fake health condition (n=59)
Physical function	0.92	0.53
Pain interference	0.95	0.76
Fatigue	0.94	0.77
Depression	0.93	0.81
Anxiety	0.92	0.80
Sleep disturbance	0.88	−0.21
Ability to participate in social roles and activities	0.94	0.78
Cognitive function	0.70	0.44

**Table 6:** Correlations among Patient-Reported Outcomes Measurement Information System-29+2 (version 2.1) preference-based score scales on the 3-month survey (did not endorse is below and endorsed fake health condition is above the diagonal).

	PF^a^	PIter^b^	PIten^c^	FAT^d^	DEP^e^	ANX^f^	SLPD^g^	SOC^h^	CF^i^
PF		−0.31	−0.38	−0.33	−0.51	−0.55	−0.25	0.32	0.12
PIter	−0.73		0.40	0.71	0.62	0.65	0.22	−0.69	0.43^j^
PIten	−0.58	0.73		0.34	0.33	0.47	0.20	−0.23	0.16^j^
FAT	−0.46	0.52	0.39		0.65	0.75	0.16	−0.71	0.33^j^
DEP	−0.33	0.40	0.33	0.61		0.82	0.26	−0.68	0.27^j^
ANX	−0.34	0.43	0.35	0.62	0.81		0.26	−0.71	0.15^j^
SLPD	−0.31	0.37	0.32	0.58	0.50	0.49		−0.26	−0.18
SOC	0.64	−0.66	−0.51	−0.69	−0.61	−0.64	−0.53		−0.17^j^
CF	0.29	−0.33	−0.30	−0.42	−0.46	−0.49	−0.38	0.49	

^a^PF: physical function.

^b^PIter: pain interference.

^c^PIten: pain intensity.

^d^FAT: fatigue.

^e^DEP: depression.

^f^ANX: anxiety.

^g^SLPD: sleep disturbance.

^h^SOC: ability to participate in social roles and activities.

^i^CF: cognitive function.

^j^Correlation is in the “wrong” direction.

**Table 7:** PROMIS-29+2^a^ (version 2.1; PROPr^b^) scale means (SDs) on the 3-month survey.

Scale	Did not endorse fake health conditions (n=972), mean (SD)	Endorsed fake health condition (n=59), mean (SD)	Overall sample (N=1031), mean (SD)
Physical function	46 (9)	41 (4)	46 (8)
Pain interference	54 (9)	62 (4)	55 (9)
Pain intensity	56 (9)	62 (9)	56 (9)
Fatigue	54 (10)	57 (7)	54 (10)
Depression	55 (10)	62 (7)	55 (10)
Anxiety	56 (10)	63 (7)	56 (10)
Sleep disturbance	53 (9)	51 (4)	53 (9)
Ability social roles or activities	51 (9)	44 (6)	51 (9)
Cognitive function	50 (8)	46 (6)	50 (8)
PROMIS-29 physical health summary	47 (9)	40 (4)	46 (9)
PROMIS-29 mental health summary	46 (9)	41 (6)	46 (9)
PROPr	0.37 (0.23)	0.22 (0.11)	0.37 (0.23)

^a^PROMIS: Patient-Reported Outcomes Measurement Information System.

^b^PROPr: PROMIS preference-based score.

Because of the small sample size of respondents who endorsed a fake condition, mean scores including and excluding those who endorsed a fake condition were similar. Note that our estimates are conservative because we estimate that there are about 4% (302/6832) “fake” respondents still undetected in the second data wave (25%-15%-6%)=4%.

## Discussion

This study provides evidence that asking about fake health conditions can help screen out respondents who may be either dishonest or careless. It shows that this subgroup of MTurk respondents differs from those who do not endorse a fake condition on demographic variables (gender, race, age, health conditions, and time to complete the survey). In addition, the minority of respondents who endorsed a fake condition provided less internally consistent responses, and their mean scores indicated the worse health-related quality of life.

The estimated 25% (1708/6832) rate of endorsing a fake health condition is consistent with prior reports of careless responses in crowdsourced samples [17]. But the rate in our study could be an underestimate because we limited the sample to those with a 95% successful completion rate on 500 previous MTurk tasks. The association of reporting a fake condition with a greater number of self-reported health conditions parallels research documenting that those who report the use of a nonexistent recreational drug (“Bindro”) tend to self-report more use of actual drugs [18].

The lower internal consistency reliability estimates in this study for those endorsing a fake health condition were likely overly optimistic because the wording of most of the items was in the same direction so consistently answering in the same direction of the response scale could bias reliability estimates upward [19]. The 1 scale (sleep disturbance) where changing the direction of responding was needed to be consistent in self-reports had zero reliability (negative α) among those who endorsed a fake condition. Note that we found a similar pattern in this data set for correlations of a PROMIS cognitive function item (“I have had trouble shifting back and forth between different activities that require thinking”) not included in PROMIS-29+2 (version 2.1) with the 2 items that are included (results not presented).

This study had several limitations. First, the sample has worse mental health, is younger, more educated, and has less income than the US general population [5]. In addition, data collection occurred during the COVID-19 pandemic. Response behavior may have differed during this time compared to that before or after the pandemic. In addition, our use of IP addresses may not have excluded some from outside the United States due to the use of virtual private networks. Another limitation is that all data in this study were self-reported. But we limited our sample to those with a history of higher-quality data. Finally, the results of this study are specific to MTurk and may not generalize to all internet panels. The motivation to be dishonest to get payment for participation is potentially reduced when money is donated to charity (eg, SurveyMonkey’s audience pool and the Opt4G internet panel) rather than directly to the respondent as a cash reimbursement.

Careless responding and acceptance acquiescent response patterns are problematic because they introduce errors in the measurement of the concept of interest [20,21]. In this study, consistently selecting extreme responses that represent worse health for most items may have been a strategy adopted by some of the MTurk respondents. Like gaming demographic questions to be study eligible, the consistent reporting of negative health may maximize the likelihood of qualifying for study participation [22]. The use of balanced scales has been advocated to address responding the same way to items regardless of content, but this means that those with problematic response patterns receive “middling scores on the scale regardless of their true attitudes” [23]. The longer length of time to complete the survey by those reporting a fake condition could be due to shared knowledge in the MTurk community that completing surveys too quickly will not be accepted as a complete task.

An important caveat about the value of including bogus health conditions to screen out respondents is that its usefulness will fade over time if information about it spreads among potential survey respondents. For example, the urban dictionary warns readers not to select “Bindro” on surveys of drug use because selecting it “voids the whole test.” If potential survey respondents become aware of the fake health conditions, it will be necessary to rely on other approaches such as consistency checks using person-fit indices for items within scales that are worded in opposite directions to identify careless respondents [24,25].

The primary implication of this study is that the quality of data collected from MTurk web-based panel members can be improved significantly by screening for careless or dishonest responses using bogus health conditions. Given that about one-fourth of the sample was estimated to belong to this subgroup of suspect respondents, researchers employing this method should plan to include about a 25% larger sample than the number of surveys they need to end up with enough completed surveys.

## Appendix

Multimedia Appendix 1 Estimated proportion of respondents who report fake conditions.

## Abbreviations

MTurk: Amazon’s Mechanical Turk
PROMIS: Patient Reported Outcomes Measurement Information System
PROPr: Patient Reported Outcomes Measurement Information System preference-based score

## Data Availability

The data set analyzed for this study is not publicly available yet because the project is still in progress, but the data are available from the first author on reasonable request.
