# Supplementary material for: Effects of Excluding Those Who Report Having “Syndomitis” or “Chekalism” on Data Quality: Longitudinal Health Survey of a Sample From Amazon’s Mechanical Turk
Source: J Med Internet Res. 2023 Aug 4;25:e46421. doi: 10.2196/46421 (PMC10439462; doi:10.2196/46421)
Supplement: Multimedia Appendix 1 [file jmir_v25i1e46421_app1.docx]

**Multimedia Appendix 1.** The estimated proportion of respondents who report fake conditions.

Assume there is a proportion *ϕ* of fake respondents in the sample. Assume that the probability to get caught (i.e., endorsing one or both fake conditions) is equal to*η.* Then in the first wave, a proportion *ϕη* of respondents will get caught. These will get removed from the sample, so in the second wave the true proportion of fake respondents will be equal to *ϕ*(1−*η*) and the proportion that will get caught in the second wave is then *ϕ*(1−*η*)*η.*

*ηϕ* = (996/6832) = .146

*ϕ*(1−*η*)*η* = (59/972) = .061

Solving these equations yields *η* = .58, *ϕ*=.25

Thus, we find that one-quarter of the respondents are fake respondents. We have assumed that the three months follow-up sample is a random draw from the non-fake respondents in the first wave.
